# Letter to editor about “The mediating effect of blood biomarkers in the associations between inflammatory bowel disease and incident psychiatric disorders: a prospective cohort study”

**DOI:** 10.1097/JS9.0000000000003373

**Published:** 2025-09-09

**Authors:** Shi-Cong Liang, Hong-Xing Li, Zhi-Chun Xia

**Affiliations:** a The Affiliated Brain Hospital, Guangzhou Medical University, Guangzhou, China; b Key Laboratory of Neurogenetics and Channelopathies of Guangdong Province and the Ministry of Education of China, Guangzhou Medical University, Guangzhou, China

*Dear Editor,*

We read with interest the recent publication examining the association between inflammatory bowel disease (IBD) and the risk of psychiatric disorders, with a particular focus on the potential mediating role of blood-based inflammatory biomarkers^[1]^. The study stands out for its ambitious scope, notably the inclusion of a broad range of psychiatric outcomes and the use of mediation analysis within a large prospective cohort. We commend the authors for their effort to bridge gastroenterology and psychiatry, contributing valuable insights into the potential biological underpinnings of the gut–brain axis. The following content complies with TITAN^[2]^.

## Validity, heterogeneity, and measurement bias in psychiatric diagnoses

The study defines psychiatric disorders based on hospital records and ICD-10 codes. While this approach ensures high specificity, it may underestimate the prevalence of milder or undiagnosed conditions, particularly in individuals without IBD^[3]^. This raises concerns about ascertainment bias, as patients with IBD due to more frequent contact with healthcare services are more likely to receive a psychiatric diagnosis^[4]^. Furthermore, the diagnostic categories themselves are highly heterogeneous. For instance, “depressive disorders” may encompass single-episode depression, recurrent depression, dysthymia, or adjustment disorders, complicating the interpretation of associated biomarkers.

## Temporal dynamics and prodromal phases of psychiatric illness

The authors attempted to reduce reverse causality by excluding cases diagnosed within the first year of follow-up a reasonable strategy. However, many psychiatric conditions, especially major depression and substance use disorders, often present with prodromal symptoms months or even years before diagnosis^[5]^. Thus, a 1-year exclusion window may not be sufficient to fully establish causal direction, particularly since common IBD-related symptoms such as fatigue, insomnia, and anhedonia may mimic or mask early manifestations of mental illness.

## Interpretation of inflammatory biomarkers: state versus trait markers

The study identifies partial mediation by inflammatory markers such as C-reactive protein (CRP), red cell distribution width (RDW), and systemic immune-inflammation index (SII) in the link between IBD and psychiatric outcomes. However, these are nonspecific systemic indicators that often fluctuate with the current physiological state, making them less reliable as stable trait biomarkers. In psychiatric research, their role in disease susceptibility or pathogenesis remains debated. For instance, CRP has been shown to rise during episodes of depression or psychosis and decrease with treatment, suggesting it may reflect disease activity or secondary stress responses rather than playing a direct causal role in psychiatric pathology^[6]^. To strengthen causal mediation claims, future work could incorporate more neuro-specific biomarkers such as neurofilament light chain, kynurenine pathway metabolites, or cytokine panels to explore mechanistic pathways more precisely.

## Conclusion

This study provides important evidence for the bidirectional link between gastrointestinal disease and mental health. From a psychiatric standpoint, we believe its explanatory power and clinical relevance would be enhanced by further attention to diagnostic accuracy, symptom trajectories, and the biological nature of inflammatory markers. We appreciate the authors’ contribution to advancing the understanding of gut–brain interactions and encourage future studies to integrate psychiatric perspectives more thoroughly in both study design and interpretation, fostering deeper interdisciplinary collaboration between gastroenterology and mental health research.

## Data Availability

Not applicable.
